# Diagnostic Performance of (1→3)-β-D-Glucan Alone and in Combination with *Aspergillus* PCR and Galactomannan in Serum of Pediatric Patients after Allogeneic Hematopoietic Stem Cell Transplantation

**DOI:** 10.3390/jof7030238

**Published:** 2021-03-22

**Authors:** Jan Springer, Jürgen Held, Carlo Mengoli, Paul Gerhardt Schlegel, Florian Gamon, Johannes Träger, Oliver Kurzai, Hermann Einsele, Juergen Loeffler, Matthias Eyrich

**Affiliations:** 1 Department for Internal Medicine II, University Hospital of Würzburg, 97080 Würzburg, Germany; florian.gamon@web.de (F.G.); Einsele_H@ukw.de (H.E.); Loeffler_J@ukw.de (J.L.); 2 Mikrobiologisches Institut—Klinische Mikrobiologie, Immunologie und Hygiene, Universitätsklinikum Erlangen und Friedrich-Alexander-Universität (FAU) Erlangen-Nürnberg, 91054 Erlangen, Germany; juergen.held@uk-erlangen.de (J.H.); johannes.traeger@fau.de (J.T.); 3 Department of Histology, Microbiology, and Medical Biotechnology, University of Padua, 35122 Padua, Italy; mengolicarlo@gmail.com; 4 Kinderklinik und Poliklinik, University Medical Center Würzburg, 97080 Würzburg, Germany; Schlegel_P@ukw.de (P.G.S.); Eyrich_M@ukw.de (M.E.); 5 National Reference Center for Invasive Fungal Infections (NRZMyk), Leibniz Institute for Natural Product Research and Infection Biology, Hans Knöll Institute, 07745 Jena, Germany; okurzai@hygiene.uni-wuerzburg.de; 6 Institute for Hygiene and Microbiology, University Würzburg, 97080 Würzburg, Germany

**Keywords:** beta-D-glucan, galactomannan, real-time PCR, *Aspergillus*, pediatric

## Abstract

Data on biomarker-assisted diagnosis of invasive aspergillosis (IA) in pediatric patients is scarce. Therefore, we conducted a cohort study over two years including 404 serum specimens of 26 pediatric patients after allogeneic hematopoietic stem cell transplantation (alloSCT). Sera were tested prospectively twice weekly for *Aspergillus*-specific DNA, galactomannan (GM), and retrospectively for (1→3)-β-D-glucan (BDG). Three probable IA and two possible invasive fungal disease (IFD) cases were identified using the European Organization for Research and Treatment of Cancer and the Mycoses Study Group (EORTC/MSGERC) 2019 consensus definitions. Sensitivity and specificity for diagnosis of probable IA and possible IFD was 80% (95% confidential interval (CI): 28–99%) and 55% (95% CI: 32–77%) for BDG, 40% (95% CI: 5–85%) and 100% (95% CI: 83–100%) for GM, and 60% (95% CI: 15–95%) and 95% (95% CI: 75–100%) for *Aspergillus-*specific real-time PCR. However, sensitivities have to be interpreted with great caution due to the limited number of IA cases. Interestingly, the low specificity of BDG was largely caused by false-positive BDG results that clustered around the date of alloSCT. The following strategies were able to increase BDG specificity: two consecutive positive BDG tests for diagnosis (specificity 80% (95% CI: 56–94%)); using an optimized cutoff value of 306 pg/mL (specificity 90% (95% CI: 68–99%)) and testing BDG only after the acute posttransplant phase. In summary, BDG can help to diagnose IA in pediatric alloSCT recipients. However, due to the poor specificity either an increased cutoff value should be utilized or BDG results should be confirmed by an alternative *Aspergillus* assay.

## 1. Introduction

(1→3)-beta-D-Glucan (BDG) is a fungal cell wall component released into patients’ blood during invasive fungal disease (IFD). BDG is not specific for *Aspergillus* infection, and could therefore be used as a broad spectrum fungal biomarker keeping in mind that it is not detectable in IFD caused by mucormycetes, *Cryptococcus* or *Blastomyces dermatitidis* [1,2]. Confounding factors leading to false positivity include bacteremia and various intravenous medications [3,4].

BDG was included as microbiological criterion in the revised consensus definitions of IFD from the European Organization for Research and Treatment of Cancer and the Mycoses Study Group (EORTC/MSG) 2008 [5], but excluded in the latest version 2019 as BDG was not considered to provide mycological evidence of any IFD [6]. Additionally, BDG is not specific for a particular IFD and consequently should not be used for classification within clinical studies. Amongst the different IFDs, invasive aspergillosis (IA) is the most significant opportunistic fungal infection in neutropenic adult and pediatric patients following allogeneic hematopoietic stem cell transplantation (alloSCT) [7]. Diagnosis of IA is challenging as clinical symptoms are often non-specific and classical diagnosis is poor [8]. Methods such as high resolution computed tomography (HRCT) scans only show typical signs once the infection is established, and even then specific signs can be transient or occur only at a very late stage of disease [8,9]. Serological tests detecting galactomannan (GM) and BDG have low positive predictive values and are better used for exclusion rather than diagnosis of IA [8,9,10]. Especially for BDG testing guidelines are missing [11]. These limitations have led to the development of PCR-based test systems to detect fungal DNA in patient specimens. However, such assays operate at the very limit of detection due to the small amounts of fungal DNA which can be recovered from blood samples. This problem is even aggravated in pediatrics where only small volumes of blood can be drawn. This bottleneck is circumvented by the Fungitell^®^ assay, which requires only five microliters of serum for a single BDG measurement. In addition to being used as a single biomarker, the combination with other assays may prove beneficial for an accurate and timely diagnosis of IA [12,13,14,15]. However, little data exists for these biomarkers (especially for BDG) in pediatric patients and even less for combined strategies. Here, we report on a highly standardized diagnostic schedule, involving systematic screening of children with a high risk of IA by BDG, GM and *Aspergillus* real-time PCR assay.

## 2. Materials and Methods

Patients. From April 2016 to March 2018 (24 months), 41 pediatric patients treated at the University Children’s Hospital Würzburg were screened twice weekly by GM and PCR-based diagnostic assays. All available patients with more than two serum samples and existence of frozen backup samples (*n* = 26) were selected for retrospective BDG analysis. Patients (12 males, median age of 11.5 years (range, 2 to 19 years); 14 females, median age of 8.5 years (range, 2 to 18 years)) were all eligible and in preparation for alloSCT. Patients suffered from the following diseases: acute lymphoblastic leukemia (*n* = 18; 69%), acute myeloid leukemia (*n* = 3; 12%), and other diseases, including neuroblastoma (*n* = 2), lymphoma, sarcoma, and immunodeficiency (*n* = 1 each). In total, 404 blood samples (mean, 15.5 samples per patient; range, 3 to 71 samples) were analysed. Routinely, all pediatric patients received liposomal amphothericin B as prophylaxis.

Clinical signs and microbiological data were recorded for each individual patient with IA defined according to the revised consensus definitions of the European Organization for the Research and Treatment of Cancer/Mycoses Study Group Education and Research Consortium (EORTC/MSGERC criteria 2019; [6]). This latest definition for IA includes PCR and GM testing as microbiological criteria, but not BDG.

For analysis of clinical test performance probable IA cases and “not classified” patients (serving as negative controls) were used including PCR and GM as microbiological criteria. Not classified patients comprise all patients except those with proven, probable and possible IFD. In a second approach also cases of possible IFD were included as true IA cases. The reason to do so was that the entity of probable IA and possible IFD cases is defined by a host factor and a clinical feature without the requirement for mycological evidence. This enabled us to compare the diagnostic performance of BDG with GM and *Aspergillus* real-time PCR without the risk of incorporation bias.

One not classified patient (K207; 18 samples) was excluded from further analysis due to a missing HRCT scan (only X-ray radiography was available showing infiltrates highly suggestive for microbial infection). Data of this patient, especially in the context of BDG detection, were analysed separately.

Diagnostic molecular assays. Patients were tested within a routine diagnostic surveillance program using GM ELISA (Platelia^TM^ *Aspergillus* Ag assay; Bio-Rad, Munich, Germany), BDG measurements and in-house fungal PCR testing. BDG measurement was performed retrospectively from serum samples that were frozen at −20 °C until testing. No additional freeze–thaw cycles occurred between GM and BDG testing. No ethical approval was necessary because BDG testing was performed retrospectively and no clinical management decisions were based on BDG results.

The Platelia^TM^ assay was performed according to the manufacturer’s recommendation using an index of ≥ 1 to define a positive GM test. A single positive test was required to define a positive GM result for EORTC/MSGERC classification.

For the *Aspergillus* PCR, DNA was extracted from a 1-mL cell-free blood fraction using the QIAamp UltraSens virus kit (Qiagen, Hilden, Germany) [16]. The protocol used was compliant with the European Aspergillus PCR Initiative (EAPCRI) recommendations for serum to guarantee the highest diagnostic standards [17]. Real-time PCR, involving an internal control, was performed as previously described [18]. Briefly, 20 μL reaction mixtures contained 0.3 μM primer Asp fum_F degen (5′-GCAGTCTGAGTTGATTATyGyAATC-3′, where y is C or T), 0.3 μM primer Fungi 5.8_R (5′-CAGGGGGCGCAATGTGC-3′), 0.15 μM hydrolysis probe ITS-PF (5′-FAM-CAGCGAAATGCGATAAGTAATGTGAATTGCA-TAMRA-3′), 10 μL of Takyon ROX Probe 2X MasterMix UNG (Eurogentec), and 5 μL of template DNA. The assay detects all clinically relevant *Aspergillus* species, but cannot differentiate between them. In a second reaction, *Bacillus* DNA used as extraction and PCR inhibition control was detected. This internal control was tested independently of the *Aspergillus* target (monoplex), but within the same PCR run. The reaction mixtures (20 μL in total) contained 0.12 μM primer 16S S (5′-ggTCTT gAC ATC CTC TgA cAA tCC TA-3′), 0.12 μM primer 16S A (5′-AAC TgA ATg CTg gCA ACT AAg ATC A-3′), 0.07 μM hydrolysis probe 16S TM (5′-FAM-AgA gTg ACA ggT ggT gCA Tgg TTg TC-TAMRA-3′), 10 μL of Takyon ROX Probe 2X MasterMix UNG, and 5 μL of template DNA. Amplification was carried out with a StepOnePlus machine (Applied Biosystems), with the following steps: 50 °C for 2 min, 95 °C for 3 min, and 60 cycles of 95 °C for 5 s, 54 °C for 15 s (detection step), and 72 °C for 1 s. Negative and positive PCR controls were included in each run, and were analysed in duplicate (*Aspergillus*). The limit of detection was below two copies. Two positive results (non-reproducible or reproducible positive duplicates) from two independent samples within 15 days were defined as positive PCR assay for EORTC/MSGERC classification.

For BDG measurement the Fungitell^®^ assay (Associates of Cape Cod, Falmouth, MA, USA) was used according to the manufacturer´s instructions. Briefly, 5 µL of serum were added to 20 µL of pretreatment reagent in a glucan-free 96-well plate. After 10 min of incubation at 37 °C, 100 µL of Fungitell^®^ reagent were added to the pretreated sample. The *Limulus* amebocyte lysate reaction was then monitored at 37 °C for 40 min in an ELx808™ microplate reader (BioTek Instruments, Winooski, VT, USA). The mean optical density change over time was calculated and compared to a standard curve to determine the BDG concentration. Samples with indeterminate (60–79 pg/mL) or positive results (≥ 80 pg/mL) were retested to confirm the elevated BDG value where possible. BDG levels below 31 pg/mL (lower validation limit) were calculated by extrapolation.

Statistics. The clinical performance, sensitivity, specificity, positive and negative predictive value (PPV and NPV, respectively), and odds ratio of the different assays were calculated using Prism 5 (Graph-Pad Software). Diagnostic odds ratio (OR) is a global measure for diagnostic accuracy. OR depends significantly on the sensitivity and specificity of a test and does not depend on disease prevalence. A test with high specificity and sensitivity with low rate of false positives and false negatives has high OR [19]. A confidence interval (CI) is a type of estimate computed from the statistics of the observed data. This proposes a range of plausible values for an unknown parameter. The interval has an associated confidence level that the true parameter is in the proposed range. The confidence level is chosen by the investigator [20]. For example, the confidence level can be 95% (95% CI). It means that confidence intervals should cover the true parameter value 95% of the time (coverage) [21], repeating the same study as before. Clearly, it expresses the uncertainty surrounding that parameter; if CI is wide, the uncertainty is large. The receiver operating characteristic (ROC) curve analysis was used to assess the diagnostic value of BDG for the diagnosis of IA. Since GM and PCR were not evaluable for the same purpose, as these assays had been employed for setting the EORTC/MSGERC state, the correlations of BDG to GM and PCR were investigated as the relations between GM and PCR. For the search of the best cutoff value, the following methods were used: maximization of the sum sensitivity + specificity, maximization of the product sensitivity x specificity, and minimization of absolute difference sensitivity—specificity.

The relations among BDG, GM, and PCR were evaluated. Grouping continuous variables by binary variables, descriptive distribution indexes were calculated. For all evaluable patients the maximum value of BDG, GM, and PCR was calculated. The following variables were created: maxbdg (continuous), maxbdgpos (binary), maxgm (binary), maxgmindex (continuous), maxgmpos2019 (binary), and maxpcrpos2019 (binary). The Kruskal–Wallis test was performed on “maxbdg” (continuous) vs. “maxgmpos2019” (binary), on “maxbdg” vs. “maxpcrpos2019” (binary), and on “maxgmindex” (continuous) vs. “maxpcrpos2019”. Maxgmpos2019 = 0 is low level (normal), maxgm = 1 is high (outside the norm). Maxpcrpos2019 = 0 is no target detection, maxpcrpos2019 = 1 is the opposite.

## 3. Results

A set of 404 serum samples from 26 pediatric patients with high-risk for IFD were collected shortly before, during and after alloSCT. These specimens were screened for the presence of fungi by using three diagnostic assays, namely, *Aspergillus*-specific real-time PCR (n = 404), GM (n = 403) and BDG assay (n = 348; mean, 13.4 samples per patient; range, 3 to 27 samples). EORTC/MSGERC classification resulted in 3 cases of probable IA (11.5%), 2 cases of possible IFD (7.7%) and 21 not classified patients without specific high-resolution CT (HRCT) scan signs of IFD (80.8%). Due to a missing HRCT scan in the time period of suspected IA one not classified patient (K207) was excluded. However, this patient was analysed separately, because he perfectly illustrates the complexity and insufficient specificity of BDG kinetics in pediatric SCT patients.

### 3.1. Clinical Performance of Biomarkers

The sensitivity, specificity, PPV and NPV of a single positive BDG measurement (cut-off ≥ 80 pg/mL) in patients with probable IA was 100% (95% CI: 29–100%), 55% (95% CI: 32–77%), 25% (95% CI: 5–57%), and 100% (95% CI: 72–100), respectively (Table 1). If two consecutive BDG-positive serum samples were required as microbiological criterion the sensitivity, specificity, PPV and NPV were 67% (95% CI: 9–99%), 80% (95% CI: 56–94%), 33% (95% CI: 4–78), and 94% (95% CI: 71–100), respectively. Appropriate values for PCR and GM are shown in Table 1, but due to incorporation bias (see methods section) not further discussed in this setting.

In order to compare the performance of BDG with GM, *Aspergillus* PCR and with combinations of these biomarkers we calculated the diagnostic parameters for probable IA and possible IFD cases versus not classified patients (Table 2). Additionally, calculations using the Kruskal–Wallis test were performed.

Combination of possible IFD patients together with probable IA patients as true IA cases reduced the sensitivity and odd ratio (OR) of all three biomarkers in general. Best sensitivity was shown for BDG (80%; 95% CI: 28–99), as one possible IFD patient was detected by BDG only, followed by PCR (60%; 95% CI: 15–95) and GM (40%; 95% CI: 5–85; Table 2). Specificity was not improved for any biomarker. GM still showed best specificity (100%; 95% CI: 83–100) and PPV (100%; 95% CI: 16–100), whereas GM and PCR performed best in OR (29 each; 95% CI: 1–749 and 2–421, respectively). When combining assays, best sensitivity was seen by any combination including BDG, best specificity, PPV and OR by the combination GM/PCR (Table 3). PPV of BDG was always below 33% (Table 1, Table 2 and Table 3), whereas for OR it can be said that in general, the higher the OR, the better the test assay.

Apparently, the combination of biomarkers did not improve the diagnostic performance if one single positive result was sufficient as microbiological criterion (Table 3). Therefore, we calculated the diagnostic performance of biomarker combinations if both biomarkers had to be positive (Table 4).

Fair sensitivity of 60% (95% CI: 17–95) was shown for the combination of BDG with PCR followed by the remaining combinations with 40% each (95% CI: 5–85). By this approach, the specificity was increased to 100% (95% CI: 83–100) for all combinations with PCR and to 95% (95% CI: 75–100) for the combination of BDG with GM.

The Kruskal–Wallis analyses showed that higher mean BDG values were associated with higher GM and PCR values, and that higher mean GM values were associated with positive PCR (Tables S1–S3 and Figure S1). However, the significance was demonstrated only for the pair BDG/GM (Kruskal–Wallis chi-squared = 5.3118, degree of freedom (df) = 1, *p*-value = 0.0212). The calculated values for PCR/BDG were close to significance (Kruskal–Wallis chi-squared = 3. 5756, df = 1, *p*-value = 0.0586), but not for PCR/GM (Kruskal–Wallis chi-squared = 2.2131, df = 1, *p*-value = 0.1368). The lack of statistical significance was likely attributable to the limited number of patients under investigation. In conclusion, all three diagnostic tests showed a trend towards higher values in cases of probable IA.

**Figure 1:**
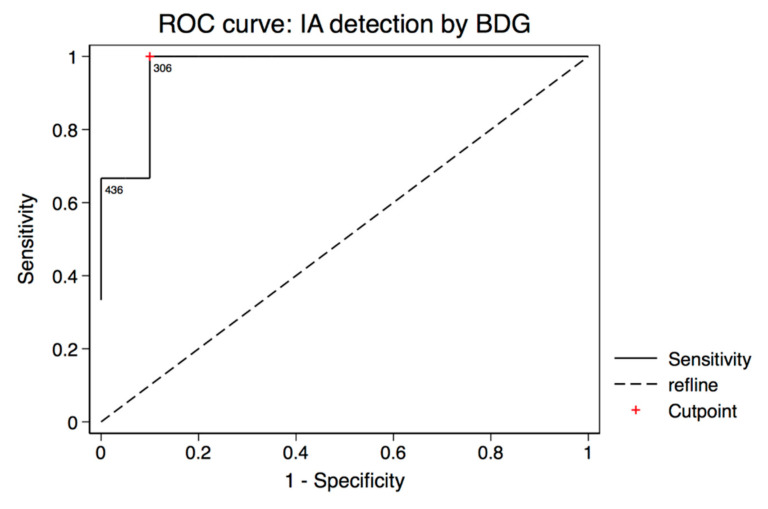
Receiver operating characteristic (ROC) curve analysis of BDG testing showing the optimal cutoff value of 306 pg/mL including probable IA cases and not classified patients as controls. Sensitivity was 100%, specificity was 90%, and the fraction of the correctly classified patients was 91%. The area under the ROC curve was 0.9667 (95% CI: 0.8874, 1).

The specificity of one single or two consecutive positive BDG measurements in our cohort was poor. In order to improve specificity, several previous studies have discussed adaptations of BDG cutoff values [22,23,24,25,26]. Therefore, we performed a ROC analysis to determine the optimal threshold in our dataset. Including probable IA cases and not classified patients as controls resulted in an optimal cutoff value at 306 pg/mL (Figure 1). At this cutoff value, BDG sensitivity was 100% (95% CI: 29–100%), specificity was 90% (95% CI: 68–99%), and the fraction of the correctly classified patients was 91%. The area under the curve was 0.9667 (95% CI: 0.8874, 1). Inclusion of the two possible IFD patients as true IA cases did not change the optimal cutoff value. The area under the ROC curve was decreased to 0.735 (95% CI: 0.4095, 1). The sensitivity was 60% (95% CI: 15–95%), the specificity was 90% (95% CI: 68–99%), and the fraction of the correctly classified patients was 84% (Figure S2).

In order to find reasons for the poor specificity of BDG we analysed the clinical data of the false-positive patients. Interestingly, 8 of 9 (89%) not classified patients with positive BDG showed condensed positivity around the date of SCT (range: 11 days before (d −11) to 49 days after SCT (d 49); Table 5).

All these patients were affected by ALL, whereas seven out of eight (88%) received a T cell depleted stem cell graft. Median age was 11 years (range: 2–17). In contrast, in a control cohort (not classified patients without BDG positivity around date of SCT, n = 6) only three patients were affected by ALL (50%) and only two received a T cell depleted graft (33%). Median age was 4.5 years (range: 2–18). In order to investigate depleted stem cells as source for BDG positivity, they were tested for the presence of BDG. The supernatant of the depleted stem cells contained 134 pg/mL BDG. However, only a very small amount is administered during SCT and consequently this cannot be responsible for systemically elevated BDG levels. Additionally, patients’ records were screened for known confounding factors of BDG positivity. Treatment with albumin or immunoglobulins and mucositis as possible sources were found in five of eight patients. Additionally, one of these patients showed signs of liver dysfunction during the observation period due to a beginning sinusoidal obstruction syndrome. Due to prophylaxis most of the patients received at least one antimicrobial drug described to be contaminated with traces of BDG [4]. Nevertheless, a clear temporal correlation between BDG positivity and the administration of these possible confounding factors could not be established. Temporal correlation of biomarkers.

**Figure 2:**
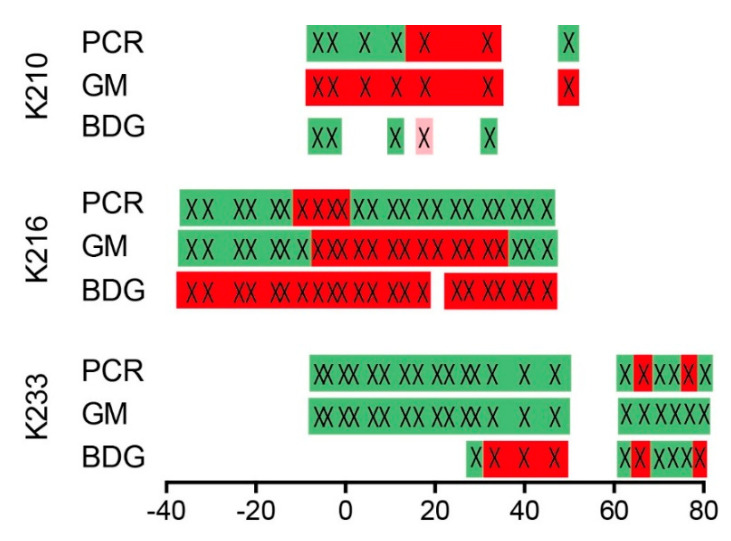
Temporal correlation of positive biomarkers (PCR, GM and BDG) in probable IA patients. The scale represents days in correlation to positive high resolution computed tomography (HRCT) scan; positive tests are indicated by crosses on red background. Green background indicates negative tests. Light red background (K210, BDG) indicates a serum with an indeterminate BDG result: 60–79 pg/mL; negative numbers indicate time before HRCT.

Biomarker positivity in probable IA cases was correlated to the time span of positive HRCT scans (Figure 2). For two probable IA cases PCR and BDG showed overlapping positivity (K216 and K233) and therefore good correlation to each other. For the third IA case (K210) an overlap was only achieved if borderline BDG results at day 17 were included (testing of one serum sample in duplicate with 80 and 71 pg/mL resulting in an indeterminate (borderline) BDG result; mean 76 pg/mL; manufacturer’s cutoff at ≥ 80 pg/mL).

### 3.2. K207—A Patient with Several Suspected Infections

**Figure 3:**
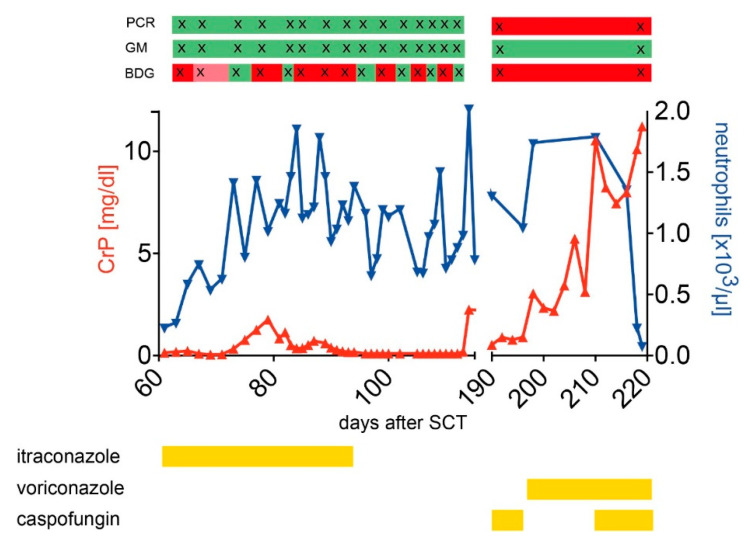
Medical history of a patient with several suspected pulmonary infections. Graph displays the course of C-reactive protein (CrP, red) and neutrophils counts (blue) over time, together with the results of the GM, PCR, and BDG assays (bars above the graph). For GM, PCR, and BDG assays, “red” indicates a positive test result, “green” a negative test result, “light red” an indeterminate result (only for BDG). Antifungal prophylaxis/treatment is outlined in yellow bars below the graph.

Patient K207 (Figure 3) was a 12-year-old boy with immunodeficiency in MHC class I complex and a confirmed mutation in the TAP1 gene. His pretransplant medical history was characterized by chronic broncho-pulmonary infections, resulting in a CF-like picture with bronchiectasis and beginning fibrosis. In addition to supportive care, regularly immunoglobulins and antibiotics, he was considered eligible for an allogeneic HSCT. After myeloablative conditioning (busulfan/fludarabine/Campath) he received a matched (10/10) unrelated donor transplant with rapid neutrophil engraftment on day 13 and an uneventful course in the early post-transplantation phase. During aplasia standard prophylaxis consisted of voriconazole, later itraconazole. Two months after alloSCT, he developed fever, cough, tachycardia, and elevated C-reactive protein (CRP) level and oxygen demand. Thorax radiography revealed progressive infiltrates in the upper lobes compatible with microbial infection. A preexisting *Stenotrophomonas maltophilia* was repeatedly detected in sputum and blood, together with other microorganisms like *Candida* species (in sputum and BAL) and viruses such as CMV and adenovirus in blood, sputum, and BAL. Although the patient partially stabilized under intensified antimicrobial treatment, the further course was characterized by an on-off situation with respiratory deterioration and partial stabilization on an intermediate care unit (IMC). On day 115 he developed an acute septicemia which necessitated mechanical ventilation and circulatory support on an ICU and treatment with voriconazole, meropenem, cefepime, and piperacillin/tazobactam (amongst other antibiotics). *Pseudomonas aeruginosa* was cultured from tracheal secretion. Under intensified regimen, the patient recovered partially, but could not be weaned entirely from mechanical ventilation support. On day 219, he died from refractory respiratory distress and multiorgan failure. Interestingly, GM was constantly negative, whereas PCR detected *A. fumigatus* at later stages of the disease between day 114 and 196 post SCT (unfortunately, no samples were available during the phase on the IMC unit). *A. fumigatus* was repeatedly cultured in tracheal secretion from day 194 onwards. HRCT scans were stopped after day 134 as they continued to show severe pulmonary alterations; however, chest X-rays repeatedly documented infectious infiltrates. Due to the lack of HRCT scans in the later phase, the patient was probably wrongly classified as “not classified”, and excluded from overall patient analysis. BDG showed positive results from the first available sample at day 65 post SCT onwards and was intermittently positive until day 114 and positive again at day 196 (next available sample). Multiple infections with *Stenotrophomonas maltophilia* and *Pseudomonas aeruginosa* as pathogens and additionally *A. fumigatus* in the terminal phase are very likely. BDG cross-reactivity with Stenotrophomonas maltophilia and *Pseudomonas aeruginosa* [27,28,29] could explain the positivity detected two months after SCT. Moreover, the regular treatment with immunoglobulins is definitely a possible source of elevated BDG levels [30]. For the final and fatal pulmonary infection, *Aspergillus fumigatus* can be regarded as the responsible agent, as this fungus was repeatedly cultured directly from tracheal secretion after day 194 until the death of the patient.

## 4. Discussion

The aim of this study was to evaluate a standardized BDG screening (twice weekly) alone and in combination with *Aspergillus* PCR and galactomannan for detection of IA in high-risk pediatric patients. In total, three molecular biomarkers (BDG, GM and *Aspergillus* DNA) were analysed in 404 blood samples of 26 pediatric patients shortly before, during, and early after alloSCT. Three cases of probable IA and 2 cases of possible IFD were classified according to EORTC/MSGERC criteria. One not classified patient was excluded and analysed separately.

During the two-year study period only three patients suffered from probable IA. This is most likely a result of the routinely administered prophylaxis with liposomal amphotericin B. The small number of IA cases in our cohort is responsible for the wide confidence intervals that flaw the results of sensitivity. Consequently, it is not feasible to compare the sensitivities of the biomarkers either alone or in combination. However, it may be mentioned that BDG was elevated in all patients with probable IA. Additionally, our results are in line with previously published results showing a very wide sensitivity range of the Fungitell assay (27% to 100%) [31].

In contrast to sensitivity, the results for specificity are based on 23 patients and more than 380 serum samples. The specificity of a single positive BDG value was only moderate but again falls into the previously published specificity range of 0% to 100% [31]. So far, only one study investigated the performance of BDG with a focus on IA in pediatric patients [3]. Badiee et al. examined 62 children from hematology–oncology and diagnosed 17 proven and probable IA cases with a specificity of 46%. In addition, two studies investigated BDG in children with IFD. First, Koltze et al. studied 34 pediatric patients after SCT with 2 proven (one fusariosis and one candidemia) and four probable IA cases and determined a specificity of 73% [10]. Second, a Chinese study on children from hematology-oncology and ICU enrolled 130 patients and diagnosed two candidemias and 20 probable cases of IFD. They calculated a specificity of 82.5% [32]. The low specificity in pediatric patients seems to be aggravated in studies investigating children with IA compared to IFD.

The low specificity of BDG is well known from adult patients and can be caused by several confounding factors including mucositis, bacterial infections or treatment with albumin, immunoglobulins or particular antimicrobial drugs [3,4,30]. At least one of these factors was present in each of our patients with false-positive BDG test results but no clear temporal correlation could be established. This proved to be difficult also in other studies [10]. Interestingly, most of the false-positive BDG patients showed clustered positivity around the date of SCT and specificity of BDG measurement increased significantly in the post-transplant period. This is not surprising considering the various treatments the patients receive in this time period. Regular administration of intravenous immunoglobulins that cause significantly elevated BDG levels after a single dose [33] or the development of mucositis with potential translocation of BDG from the gut into the blood stream are just two explanations. As a consequence, careful interpretation of BDG results in this time period is mandatory.

What are ways to increase specificity in pediatric patients after SCT? One possibility is the requirement of a second consecutive positive BDG test. In our cohort the specificity increased from 55 to 80% by this measure. Even stronger is the gain in specificity if BDG is combined with PCR and both biomarkers have to be positive. Specificity is 100% with this approach and three out of five patients with probable IA and possible IFD are detected (sensitivity 60%). Another possibility is to increase the cutoff. Adapting the BDG threshold for IFD was discussed for adults [10,25] and very recently for pediatric patients [26]. Calitri et al. evaluated different cutoffs in children up to 400 pg/mL. Good specificity (>90%) was reached above 200 pg/mL, whereas the best balance between sensitivity and specificity was achieved near 350 pg/mL [26]. This is very similar to the value calculated by our ROC curve analysis (306 pg/mL), but is in clear contrast to BDG cutoffs either reviewed using the EORTC/MSG criteria as a reference standard (60 pg/mL; [25]) or recommended by the manufacturer (80 pg/mL). A reason for the discrepancy between adult and pediatric cutoff values could be different baseline BDG levels. In fact, Smith et al. showed that pediatric patients have a one-third higher background level of BDG in comparison to adults and consequently cutoff values have to be higher in children [22]. Interestingly, for GM a very similar sensitivity and specificity has been reported in adults and children [34].

Another approach is to exclude BDG completely from screening around the day of SCT. This would increase the specificity of the whole assay tremendously. During this time, clinical signs together with biomarker combinations could be used to screen for IFD. According to our data a combination of BDG or GM with *Aspergillus*-specific PCR is able to detect probable IA and possible IFD with excellent specificity (100% and 95%, respectively) and PPV (100% and 75%, respectively). Therefore, BDG or GM and *Aspergillus*-specific PCR could be used during the SCT phase.

As BDG is not specific for *Aspergillus*, the test can be used to detect other fungi, but cross-reacts also with some bacteria [27,28,29]. Cross-reactivity with microbial antigens could also explain the detection of elevated BDG levels in one possible IFD patient and explain why the more *Aspergillus*-specific assays GM or PCR had not detected this patient. Another case of false-positivity is intensively described above (K207), as this patient suffered from different bacterial infections showing intermittently numerous positive BDG results.

A clear limitation of our study is the limited number of patients in our study cohort resulting in a low number of probable IA cases. Due to a limited number of probable IA cases the CI of sensitivity was quite wide for all biomarkers making conclusions difficult. In contrast, results for specificity and NPV showed a relatively narrow CI confirming the high value of NPV. Future multicenter studies are necessary to finally solve this issue in pediatric patients. As this is a single-center study and only pediatric patients were included, our data represent another brick in the wall of evidences about the applicability of BDG, especially in respect to the pediatric field where data is quite scarce. Our results are in accordance with other published data describing low PPV for BDG screening if used as sole biomarker and therefore limited usefulness as screening tool [10]. On the other hand, BDG can be used in combination with PCR and as additional diagnostic tool, especially in the post-acute screening phase after alloSCT.

## 5. Conclusions

BDG can help to diagnose IA in pediatric SCT recipients when used in combination with PCR and as a sole biomarker in the surveillance phase of alloSCT patients after mucositis has resolved and intense antimicrobial treatments are not required any more. Specificity can be increased significantly by raising the cutoff and by the requirement for two consecutive positive BDG results.

## Figures and Tables

**Table 1:** Clinical performance in probable invasive aspergillosis (IA) cases. PPV and NPV, positive and negative predictive value, respectively; OR, odds ratio; CI, confidential interval; 2x (1→3)-beta-D-Glucan (BDG), 2 consecutive tests were considered as positive.

	% (95% CI)			
	qPCR	GM	BDG	2x BDG
Sensitivity	100 (29–100)	67 (9–99)	100 (29–100)	67 (9–99)
Specificity	95 (75–100)	100 (83–100)	55 (32–77)	80 (56–94)
PPV	75 (19–99)	100 (16–100)	25 (5–57)	33 (4–78)
NPV	100 (81–100)	95 (76–100)	100 (72–100)	94 (71–100)
OR	91 (3–2720)	68 (2–2175)	8 (0–186)	8 (1–112)

**Table 2:** Clinical performance in probable IA and possible IFD cases. PPV and NPV, positive and negative predictive value, respectively; OR, odds ratio; CI, confidential interval; 2x BDG, 2 consecutive tests were considered as positive.

	% (95% CI)			
	qPCR	GM	BDG	2x BDG
Sensitivity	60 (15–95)	40 (5–85)	80 (28–99)	40 (5–86)
Specificity	95 (75–100)	100 (83–100)	55 (32–77)	80 (56–94)
PPV	75 (19–99)	100 (16–100)	31 (9–61)	33 (4–78)
NPV	90 (70–99)	87 (66–97)	92 (62–100)	84 (60–97)
OR	29 (2–421)	29 (1–749)	5 (0–52)	3 (0–22)

**Table 3:** Clinical performance of different assay combinations in probable IA and possible IFD cases. PPV and NPV, positive and negative predictive value, respectively; OR, odds ratio; CI, confidential interval.

	% (95% CI)			
	qPCR/GM	PCR/BDG	GM/BDG	PCR/GM/BDG
Sensitivity	60 (15–95)	80 (28–99)	80 (28–99)	80 (28–99)
Specificity	95 (75–100)	50 (27–73)	55 (32–77)	50 (27–73)
PPV	75 (19–99)	29 (8–58)	31 (9–61)	29 (8–58)
NPV	90 (70–99)	91 (59–100)	92 (62–100)	91 (59–100)
OR	29 (2–421)	4 (0–42)	5 (0–52)	4 (0–42)

**Table 4:** Clinical performance of different assay combinations in probable IA and possible IFD cases if both/all biomarkers had to be positive. PPV and NPV, positive and negative predictive value, respectively; OR, odds ratio; CI, confidential interval.

	% (95% CI)			
	BDG/PCR	BDG/GM	GM/PCR	BDG/GM/PCR
Sensitivity	60 (17–95)	40 (5–85)	40 (5–85)	40 (5–85)
Specificity	100 (83–100)	95 (75–100)	100 (83–100)	100 (83–100)
PPV	100 (29–100)	67 (9–99)	100 (16–100)	100 (16–100)
NPV	91 (71–99)	86 (65–97)	87 (66–97)	87 (66–97)
OR	57 (2–1468)	13 (1–187)	29 (1–749)	29 (1–749)

**Table 5:** Features of not classified patients with positive BDG testing. a: d, days in correlation to SCT; negative numbers indicate time before SCT. SC(T), stem cell (transplantation).

Patient	Pos. BDG (d) a	No. of Pos. BDG	Possible Source	Disease	Depleted SC	Age
K094	−9, 6 to 39, 49	12	mucositis, albumin	ALL	yes	11
K229	9, 14	2	mucositis	ALL	no	16
K231	7 to 18	4	unknown	ALL	yes	9
K237	−1	1	unknown	ALL	yes	6
K246	−2	1	immunoglobulins	ALL	yes	2
K250	−11, −4, 5 to 19	7	mucositis	ALL	yes	13
K256	−1, 17	2	mucositis, immunoglobulins	ALL	yes	17
K260	−2, 5, 14, 17, 21, 28	6	unknown	ALL	yes	11

## Supplementary Materials

The following are available online at https://www.mdpi.com/2309-608X/7/3/238/s1: Figure S1: Matrix of scatterplots indicating the relations between three diagnostic tests. Figure S2. ROC curve analysis of BDG testing showing the optimal cutoff value of 306 pg/ml including probable IA/possible IFD cases and not classified patients as controls. Sensitivity was 60%, specificity was 90%, and the fraction of the correctly classified patients was 84%. The area under the curve was 0.735 (95% CI: 0.4095, 1). Table S1: Grouping of patients by maxGM index reporting main summary indexes of maxbdg. Table S2. Grouping of patients by maxPCR2019 index reporting main summary indexes of maxBDG. Table S3. Grouping of patients by maxPCR2019 index reporting main summary indexes of maxGM.
