# Supplementary material for: Diagnostic Performance of (1→3)-β-D-Glucan Alone and in Combination with Aspergillus PCR and Galactomannan in Serum of Pediatric Patients after Allogeneic Hematopoietic Stem Cell Transplantation
Source: J Fungi (Basel). 2021 Mar 22;7(3):238. doi: 10.3390/jof7030238 (PMC8004996; doi:10.3390/jof7030238)
Supplement: Supplementary file 1 [file jof-07-00238-s001.zip › Suppl BDG child_v2.pdf]

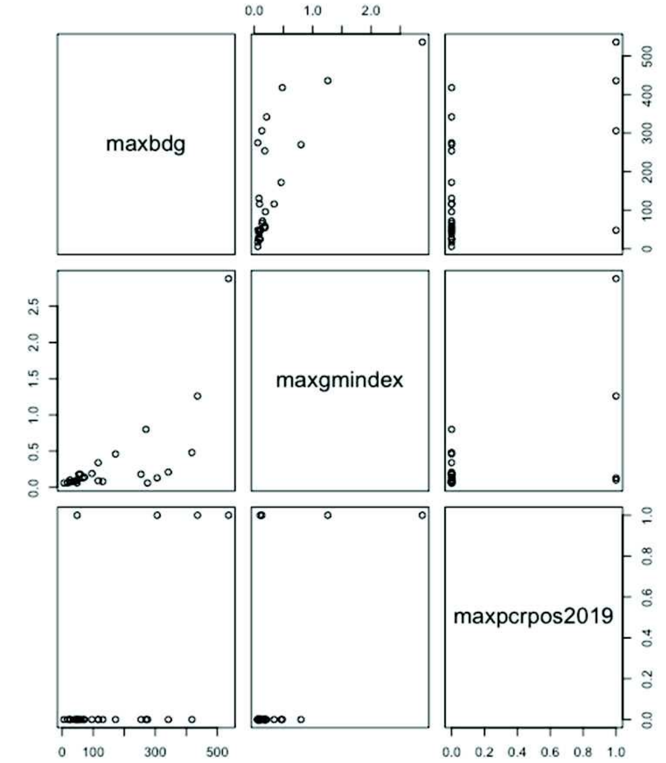

Figure. S1. Matrix of scatterplots indicating the relations between three diagnostic tests.

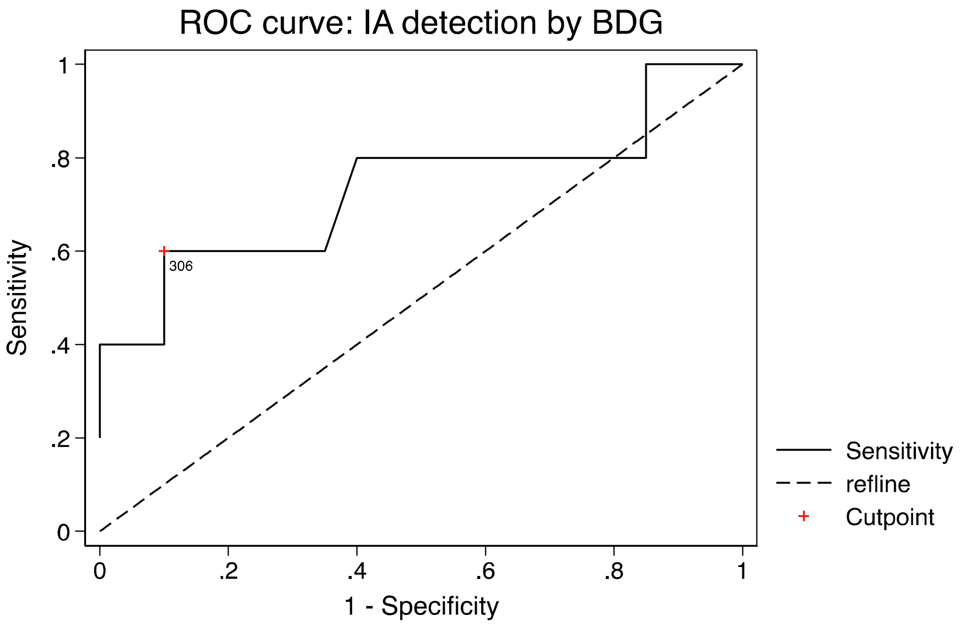

Fig. S2. ROC curve analysis of BDG testing showing the optimal cutoff value of 306 pg/ml including probable IA/possible IFD cases and not classified patients as controls. Sensitivity was 60%, specificity was 90%, and the fraction of the correctly classified patients was 84%. The area under the curve was 0.735 (95% CI: 0.4095, 1).

Table S1. Grouping of patients by maxGM index reporting main summary indexes of maxbdg

maxGMpos2019 a	patients	mean	sd	median	IQR
0	23	130	120	72	166
1	2	486	70.7	486	50

a: Maxgmpos2019 = 0 is low level (normal), maxgm = 1 is high (outside the norm).
standard deviation (sd), interquartile range (IQR)

Table S2. Grouping of patients by maxPCR2019 index reporting main summary indexes of maxBDG.

maxPCRpos2019 a	patients	mean	sd	median	IQR
0	21	126	118	72	127
1	4	332	211	371	220

a: Maxpcrpos2019 = 0 is no target detection, maxpcrpos2019 = 1 is the opposite.
standard deviation (sd), interquartile range (IQR)

Table S3. Grouping of patients by maxPCR2019 index reporting main summary indexes of maxGM.

maxPCRpos2019 a	patients	mean	sd	median	IQR
0	21	0.192	0.186	0.13	0.11
1	4	1.09	1.31	0.695	1.54

a: Maxpcrpos2019 = 0 is no target detection, maxpcrpos2019 = 1 is the opposite.
standard deviation (sd), interquartile range (IQR)
