# Supplementary material for: Diagnostic Performance of (1→3)-β-D-Glucan Alone and in Combination with Aspergillus PCR and Galactomannan in Serum of Pediatric Patients after Allogeneic Hematopoietic Stem Cell Transplantation
Source: J Fungi (Basel). 2021 Mar 22;7(3):238. doi: 10.3390/jof7030238 (PMC8004996; doi:10.3390/jof7030238)
Supplement: Supplementary file 1 [file jof-07-00238-s001.zip › Biomarker BDG child_v5.doc]

Article

**Beta-D-Glucan beside PCR and Galactomannan for Diagnosis of Invasive Aspergillosis in Serum of Pediatric Patients**

Jan Springer1*****,#, Jürgen Held2#, Carlo Mengoli3, Paul Gerhardt Schlegel4, Florian Gamon1, Johannes Träger2, Oliver Kurzai5,6, Hermann Einsele1, Juergen Loeffler1, Matthias Eyrich4

1	Department for Internal Medicine II, University Hospital of Würzburg, 97080 Würzburg, Germany

2	Mikrobiologisches Institut - Klinische Mikrobiologie, Immunologie und Hygiene; Universitätsklinikum Erlangen und Friedrich-Alexander-Universität (FAU) Erlangen-Nürnberg, 91054 Erlangen, Germany

3	Department of Histology, Microbiology, and Medical Biotechnology, University of Padua, Padua, Italy

4	Kinderklinik und Poliklinik, University Medical Center Würzburg, 97080 Würzburg, Germany

5	National Reference Center for Invasive Fungal Infections (NRZMyk), Leibniz Institute for Natural Product Research and Infection Biology, Hans Knöll Institute, 07745 Jena, Germany

6	Institute for Hygiene and Microbiology, University Würzburg; 97080 Würzburg, Germany

*****	Correspondence:	[springer_j(add)ukw.de](mailto:springer_j@ukw.de); Tel. +49-931-201-36404. Fax: +49-931-201-36409

\#	Authors contributed equally.

**Abstract**

Data on biomarker-assisted diagnosis of invasive aspergillosis (IA) in pediatric patients is scarce. Therefore, we conducted a cohort study over two years including 404 serum specimens of 26 pediatric patients after allogeneic haematopoietic stem cell transplantation (alloSCT). Sera were tested twice weekly for *Aspergillus*-specific DNA, galactomannan (GM) and (1⭢3)-β-D-glucan (BDG). Three probable IA and two possible invasive fungal disease (IFD) cases were identified using the EORTC/MSGERC 2019 consensus definitions. Sensitivity and specificity for diagnosis of probable IA and possible IFD was 80% and 55% for BDG, 60% and 100% for GM and 80% and 95% for *Aspergillus-*specific real-time PCR. Interestingly, the low specificity of BDG was largely caused by false-positive BDG results that clustered around the date of alloSCT. The following strategies were able to increase BDG specificity: two consecutive positive BDG tests for diagnosis (specificity 80%, sensitivity 40%); using an optimized cutoff value of 304 pg/ml (specificity 80%, sensitivity 60%) and testing BDG only after the acute posttransplant phase. In summary, BDG can help to diagnose IA in pediatric alloSCT recipients. However, due to the poor specificity either an increased cutoff value should be utilized or BDG results should be confirmed by an alternative *Aspergillus* assay.

**Keywords: beta-D-glucan, galactomannan, real-time PCR, *Aspergillus*, pediatric**

**1. Introduction**

(1⭢3)-beta-D-Glucan (BDG) is a fungal cell wall component released into patients’ blood during invasive fungal disease (IFD). BDG is not specific for *Aspergillus* infection, and could therefore be used as a broad spectrum fungal biomarker keeping in mind that it is not detectable in IFD caused by mucormycetes, *Cryptococcus* or *Blastomyces dermatitidis* . Confounding factors leading to false positivity include bacteremia and various intravenous medications .

BDG was included as microbiological criterion in the revised consensus definitions of IFD from the European Organization for Research and Treatment of Cancer and the Mycoses Study Group (EORTC/MSG) 2008 , but excluded in the latest version 2019 as BDG was not considered to provide mycological evidence of any IFD . Additionally, BDG is not specific for a particular IFD and consequently should not be used for classification within clinical studies. Amongst the different IFDs, invasive aspergillosis (IA) is the most significant opportunistic fungal infection in neutropenic adult and pediatric patients following allogeneic haematopoietic stem cell transplantation (alloSCT) . Diagnosis of IA is challenging as clinical symptoms are often non-specific and classical diagnosis is poor . Methods such as high resolution computed tomography (HRCT) scans only show typical signs once the infection is established, and even then specific signs can be transient or occur only at a very late stage of disease . Serological tests detecting galactomannan (GM) and BDG have low positive predictive values and are better used for exclusion rather than diagnosis of IA . Especially for BDG testing guidelines are missing . These limitations have led to the development of PCR-based test systems to detect fungal DNA in patient specimens. However, such assays operate at the very limit of detection due to the small amounts of fungal DNA which can be recovered from blood samples. This problem is even aggravated in pediatrics where only small volumes of blood can be drawn. This bottleneck is circumvented by the Fungitell® assay, which requires only five microliters of serum for a single BDG measurement. Besides being used as a single biomarker, the combination with other assays may prove beneficial for an accurate and timely diagnosis of IA . However, little data exists for these biomarkers (especially for BDG) in pediatric patients and even less for combined strategies. Here, we report on a highly standardized diagnostic schedule, involving systematic screening of children with a high risk of IA by BDG, GM and *Aspergillus* real-time PCR assay.

**2. MATERIALS AND METHODS**

**Patients.** From April 2016 to March 2018 (24 month), 41 pediatric patients treated at the University Children’s Hospital Würzburg were screened twice weekly by GM and PCR-based diagnostic assays. All available patients with more than two serum samples and existence of frozen backup samples (*n*=26) were selected for retrospective BDG analysis. Patients (12 males, median age of 11.5 years [range, 2 to 19 years]; 14 females, median age of 8.5 years [range, 2 to 18 years]) were all eligible and in preparation for alloSCT. Patients suffered from the following diseases: acute lymphoblastic leukemia (n=18; 69%), acute myeloid leukemia (*n=*3; 12%), and other diseases, including neuroblastoma (n=2), lymphoma, sarcoma, and immunodeficiency (*n=*1 each). In total, 404 blood samples (mean, 15.5 samples per patient; range, 3 to 71 samples) were analysed.

Clinical signs and microbiological data were recorded for each individual patient with IA defined according to the revised consensus definitions of the European Organization for the Research and Treatment of Cancer/Mycoses Study Group Education and Research Consortium (EORTC/MSGERC criteria 2019; ). This latest definition for IA includes PCR and GM testing as microbiological criteria, but not BDG. For analysis of clinical test performance probable IA cases and “not classified” patients (serving as negative controls) were used including PCR and GM as microbiological criteria. Not classified patients comprise all patients except those with proven, probable and possible IFD. In a second approach also cases of possible IFD were included as true IA cases. The reason to do so was that the entity of probable IA and possible IFD cases is defined by a host factor and a clinical feature without the requirement for mycological evidence. This enabled us to compare the diagnostic performance of BDG with GM and *Aspergillus* real-time PCR without the risk of incorporation bias.

One not classified patient (K207; 18 samples) was excluded from further analysis due to a missing HRCT scan (only X-ray radiography was available showing infiltrates highly suggestive for microbial infection). Data of this patient, especially in the context of BDG detection, were analysed separately.

**Diagnostic molecular assays.** Patients were tested within a routine diagnostic surveillance program using GM ELISA (Platelia^TM^ *Aspergillus* Ag assay; Bio-Rad, Munich, Germany) and in-house fungal PCR testing. BDG measurement was performed retrospectively from serum samples that were frozen at -20 °C until testing. No additional freeze-thaw cycles occurred between GM and BDG testing.

The Platelia^TM^ assay was performed according to the manufacturer´s recommendation using an index of >=1 to define a positive GM test. A single positive test was required to define a positive GM result for EORTC/MSGERC classification.

For the *Aspergillus* PCR, DNA was extracted from a 1-ml cell-free blood fraction using the QIAamp UltraSens virus kit (Qiagen, Hilden, Germany) . The protocol used was compliant with the European Aspergillus PCR Initiative (EAPCRI) recommendations for serum to guarantee the highest diagnostic standards . Real-time PCR, involving an internal control, was performed as previously described . Two positive results (non-reproducible or reproducible positive duplicates) from two independent samples within 15 days were defined as positive PCR assay for EORTC/MSGERC classification.

For BDG measurement the Fungitell^®^ assay (Associates of Cape Cod, USA) was used according to the manufacturer´s instructions. Briefly, 5 µl of serum were added to 20 µl of pretreatment reagent in a glucan-free 96-well plate. After 10 min of incubation at 37 °C, 100 µl of Fungitell^®^ reagent were added to the pretreated sample. The *Limulus* amebocyte lysate reaction was then monitored at 37 °C for 40 min in an ELx808™ microplate reader (BioTek Instruments, Winooski, USA). The mean optical density change over time was calculated and compared to a standard curve to determine the BDG concentration. Samples with indeterminate (60-79 pg/ml) or positive results (>= 80 pg/ml) were retested to confirm the elevated BDG value where possible. BDG levels below 31 pg/ml (lower validation limit) were calculated by extrapolation.

**Statistics.** The clinical performance, sensitivity, specificity, positive and negative predictive value (PPV and NPV, respectively), and odds ratio of the different assays were calculated using Prism 5 (Graph-Pad Software). The receiver operating characteristic (ROC) curve analysis was used to assess the diagnostic value of BDG for the diagnosis of IA. Since GM and PCR were not evaluable for the same purpose, as these assays had been employed for setting the EORTC/MSGERC state, the correlations of BDG to GM and PCR were investigated as the relations between GM and PCR. For the search of the best cutoff value, the following methods were used: maximization of the sum sensitivity + specificity, maximization of the product sensitivity x specificity, and minimization of absolute difference sensitivity – specificity.

The relations among BDG, GM, and PCR were evaluated. Grouping continuous variables by binary variables, descriptive distribution indexes were calculated. For all evaluable patients the maximum value of BDG, GM, and PCR was calculated. The following variables were created: maxbdg (continuous), maxbdgpos (binary), maxgm (binary), maxgmindex (continuous), maxgmpos2019 (binary), maxpcrpos2019 (binary). The Kruskal-Wallis test was performed on “maxbdg” (continuous) vs “maxgmpos2019” (binary), on “maxbdg” vs “maxpcrpos2019” (binary), and on “maxgmindex” (continuous) vs “maxpcrpos2019”. Maxgmpos2019 = 0 is low level (normal), maxgm = 1 is high (outside the norm). Maxpcrpos2019 = 0 is no target detection, maxpcrpos2019 = 1 is the opposite.

**3. Results**

A set of 404 serum samples from 26 pediatric patients with high-risk for IFD were collected shortly before, during and after alloSCT. These specimens were screened for the presence of fungi by using three diagnostic assays, namely *Aspergillus*-specific real-time PCR (n=404), GM (n=403) and BDG assay (n=348; mean, 13.4 samples per patient; range, 3 to 27 samples). EORTC/MSGERC classification resulted in 3 cases of probable IA (11.5%), 2 cases of possible IFD (7.7%) and 21 not classified patients without specific high-resolution CT (HRCT) scan signs of IFD (80.8%). Due to a missing HRCT scan and bacterial infections one not classified patient (K207) was excluded and his BDG kinetics was analysed separately.

**Clinical performance of biomarkers**

As shown in Table 1, all three probable IA cases were detected by PCR and BDG generating 100% sensitivity. The GM assay detected only two cases resulting in 67% sensitivity, but reached best specificity and PPV (100% each). All three assays reached very good NPV (95 to 100%). As BDG performed not satisfactory in specificity (55%), a second consecutive positive test was used to determine BDG positivity. As expected, this reduced sensitivity (67%) and increased specificity (80%; Table 1).

**Table 1**. Clinical performance in probable IA cases. PPV and NPV, positive and negative predictive value, respectively; OR, odds ratio; CI, confidential interval; 2x BDG, 2 consecutive tests were considered as positive. Values showing best performance are in boldface.

|  | % (95% CI) | | | |
| --- | --- | --- | --- | --- |
|  | qPCR | GM | BDG | 2x BDG |
| Sensitivity | **100** (29-100) | 67 (9-99) | **100** (29-100) | 67 (9-99) |
| Specificity | 95 (75-100) | **100** (83-100) | 55 (32-77) | 80 (56-94) |
| PPV | 75 (19-99) | **100** (16-100) | 25 (5-57) | 33 (4-78) |
| NPV | **100** (81-100) | 95 (76-100) | **100** (72-100) | 94 (71-100) |
| OR | **91** (3-2720) | 68 (2-2175) | 8 (0-186) | 8 (1-112) |

Notably, combining all three assays did not improve the results. Best performance was calculated for the combination PCR/GM (Table 2). However, as mentioned in the methods section, PCR and GM were used for EORTC/MSGERC classification. Therefore, their diagnostic performance must not be compared directly with that of BDG in order to avoid incorporation bias.

**Table 2**. Clinical performance of assay combinations in probable IA cases. PPV and NPV, positive and negative predictive value, respectively; OR, odds ratio; CI, confidential interval. Values showing best performance are in boldface.

|  | % (95% CI) | | | |
| --- | --- | --- | --- | --- |
|  | qPCR/GM | PCR/BDG | GM/BDG | PCR/GM/BDG |
| Sensitivity | **100** (29-100) | **100** (29-100) | **100** (29-100) | **100** (29-100) |
| Specificity | **95** (75-100) | 50 (27-73) | 55 (32-77) | 50 (27-73) |
| PPV | **75** (19-99) | 23 (5-54) | 25 (5-57) | 23 (5-54) |
| NPV | **100** (82-100) | **100** (69-100) | **100** (72-100) | **100** (69-100) |
| OR | **91** (3-2720) | 7 (0-153) | 8 (0-186) | 7 (0-153) |

Interestingly, 8 of 9 (89%) not classified patients with positive BDG showed condensed positivity around the date of SCT (range: 12 days before (d -12) to 49 days after SCT (d 49); Table 3).

**Table 3**. Features of not classified patients with positive BDG testing. a: d, days in correlation to SCT; negative numbers indicate time before SCT. SC(T), stem cell (transplantation).

| **patient** | **pos. BDG (d) a** | **no. of pos. BDG** | **possible source** | **disease** | **depleted SC** | **age** |
| --- | --- | --- | --- | --- | --- | --- |
| K094 | -9, 6 to 39, 49 | 12 | mucositis, albumin | ALL | yes | 11 |
| K229 | 9, 14 | 2 | mucositis | ALL | no | 16 |
| K231 | 7 to 18 | 4 | unknown | ALL | yes | 9 |
| K237 | -1 | 1 | unknown | ALL | yes | 6 |
| K246 | -2 | 1 | immunoglobulins | ALL | yes | 2 |
| K250 | -11, -4, 5 to 19 | 7 | mucositis | ALL | yes | 13 |
| K256 | -1, 17 | 2 | mucositis, immunoglobulins | ALL | yes | 17 |
| K260 | -2, 5, 14, 17, 21, 28 | 6 | unknown | ALL | yes | 11 |

All these patients were affected by ALL, whereas 7 out of 8 (88%) received a T cell depleted stem cell graft. Median age was 11 years (range: 2-17). In contrast, in a control cohort (not classified patients without BDG positivity around date of SCT, n=6) only three patients were affected by ALL (50%) and only two received a T cell depleted graft (33%). Median age was 4.5 years (range: 2-18). In order to investigate depleted stem cells as source for BDG positivity, they were tested for the presence of BDG. The supernatant of the depleted stem cells contained 134 pg/ml BDG. However, only a very small amount is administered during SCT and consequently this cannot be responsible for systemically elevated BDG levels. Additionally, patients’ records were screened for known confounding factors of BDG positivity. Treatment with albumin or immunoglobulins and mucositis as possible sources were found in five of eight patients. Due to prophylaxis most of the patients received at least one antimicrobial drug described to be contaminated with traces of BDG . Nevertheless, a clear temporal correlation between BDG positivity and the administration of these possible confounding factors could not be established.

Adaptations of BDG cutoff values were discussed in various other publications . Therefore, we performed a ROC analysis to determine the optimal threshold in our dataset. Including probable IA cases and not classified patients as controls resulted in an optimal cutoff value at 306 pg/ml (Fig. 1). At this cutoff value, BDG sensitivity was 100%, specificity was 90%, and the fraction of the correctly classified patients was 91%. The area under the curve was 0.9667 (95% CI: 0.8874, 1). Inclusion of the two possible IFD patients as true IA cases did not change the optimal cutoff value. The area under the ROC curve was decreased to 0.735 (95% CI: 0.4095, 1). The sensitivity was 60%, the specificity was 90%, and the fraction of the correctly classified patients was 84% (Fig. S2).

**Figure 1**. ROC curve analysis of BDG testing showing the optimal cutoff value of 306 pg/ml including probable IA cases and not classified patients as controls. Sensitivity was 100%, specificity was 90%, and the fraction of the correctly classified patients was 91%. The area under the ROC curve was 0.9667 (95% CI: 0.8874, 1).

The latest EORTC/MSGERC classification of probable IA cases includes *Aspergillus* PCR and GM as microbiological criteria . To calculate their diagnostic performance on probable versus not classified IA patients would introduce a strong incorporation bias. However, comparison of probable plus possible IFD patients with not classified patients is free of incorporation bias, because probable plus possible IFD patients need only a host factor and a clinical feature and no microbiological criterion (i.e. GM or *Aspergillus* PCR). Therefore, possible IFD cases were included in a second analysis (Table 4) and calculations using the Kruskal-Wallis test were performed. Inclusion of possible IFD patients together with probable IA patients as true IA cases reduced the sensitivity and odd ratio (OR) of all three biomarkers in general. Best sensitivity was shown for BDG (80%), as one possible IFD patient was detected by BDG only, followed by PCR (60%) and GM (40%; Table 4). Specificity was not improved for any biomarker. GM still showed best specificity (100%) and PPV (100%), whereas GM and PCR performed best in OR (29 each). When combining assays, best sensitivity was seen by any combination including BDG, best specificity, PPV and OR by the combination GM/PCR (Table 5). PPV of BDG was always below 33% (Table 1, 2, 4 and 5).

**Table 4**. Clinical performance in probable IA and possible IFD cases. PPV and NPV, positive and negative predictive value, respectively; OR, odds ratio; CI, confidential interval; 2x BDG, 2 consecutive tests were considered as positive. Values showing best performance are in boldface.

|  | % (95% CI) | | | |
| --- | --- | --- | --- | --- |
|  | qPCR | GM | BDG | 2x BDG |
| Sensitivity | 60 (15-95) | 40 (5-85) | **80** (28-99) | 40 (5-86) |
| Specificity | 95 (75-100) | **100** (83-100) | 55 (32-77) | 80 (56-94) |
| PPV | 75 (19-99) | **100** (16-100) | 31 (9-61) | 33 (4-78) |
| NPV | 90 (70-99) | 87 (66-97) | **92** (62-100) | 84 (60-97) |
| OR | **29** (2-421) | **29** (1-749) | 5 (0-52) | 3 (0-22) |

**Table 5**. Clinical performance of different assay combinations in probable IA and possible IFD cases. PPV and NPV, positive and negative predictive value, respectively; OR, odds ratio; CI, confidential interval. Values showing best performance are in boldface.

|  | % (95% CI) | | | |
| --- | --- | --- | --- | --- |
|  | qPCR/GM | PCR/BDG | GM/BDG | PCR/GM/BDG |
| Sensitivity | 60 (15-95) | **80** (28-99) | **80** (28-99) | **80** (28-99) |
| Specificity | **95** (75-100) | 50 (27-73) | 55 (32-77) | 50 (27-73) |
| PPV | **75** (19-99) | 29 (8-58) | 31 (9-61) | 29 (8-58) |
| NPV | 90 (70-99) | 91 (59-100) | **92** (62-100) | 91 (59-100) |
| OR | **29** (2-421) | 4 (0-42) | 5 (0-52) | 4 (0-42) |

The Kruskal-Wallis analyses showed that higher mean BDG values were associated with higher GM and PCR values, and that higher mean GM values were associated with positive PCR (Table S1-S3 and Fig. S1). However, the significance was demonstrated only for the pair BDG/GM (Kruskal-Wallis chi-squared = 5.3118, degree of freedom (df) = 1, p-value = 0.0212). The calculated values for PCR/BDG were close to significance (Kruskal-Wallis chi-squared = 3. 5756, df = 1, p-value = 0.0586), but not for PCR/GM (Kruskal-Wallis chi-squared = 2.2131, df = 1, p-value = 0.1368). The lack of statistical significance was likely attributable to the limited number of patients under investigation. In conclusion, all three diagnostic tests showed a trend towards higher values in cases of probable IA.

**Temporal correlation of biomarkers**

Biomarker positivity in probable IA and possible IFD cases was correlated to the time span of positive HRCT scans and for BDG also in correlation to the day of SCT (Table 6). For two probable IA cases PCR and BDG showed overlapping positivity (K216 and K233, Table 6, bold characters) and therefore good correlation to each other. For the third IA case (K210) an overlap was only achieved if borderline BDG results at day 17 were included (d **[17]**; testing of one serum sample in duplicate with 80 and 71 pg/ml resulting in an indeterminate (borderline) BDG result; mean 76 pg/ml; manufacturer’s cutoff at >=80 pg/ml). Other positive BDG samples of this patient showed no temporal correlation to HRCT scans (day -181 and 353).

Table 6 also illustrates the relation of BDG positivity to the date of SCT (last column). BDG positivity occurred frequently around SCT, as well as 200 and 500 days post SCT. For patient K216 BDG positivity around SCT was confirmed by positive PCR and GM results. Also one possible IFD case (K235) showed BDG positivity around SCT.

**Table 6**. Temporal correlation of positive biomarkers. The numbers represent days in correlation to positive HRCT scan; numbers in square brackets indicate a serum with an indeterminate BDG result: 60-79 pg/ml; negative numbers indicate time before HRCT/SCT. (X,Y): time point of two requested positive PCR results; if second PCR result is not stated, time to second positive PCR result is 3-4 days. a: no sample in between available; b: both PCR amplicons reveal DNA of *A. flavus*; bold numbers indicate positive samples of at least two biomarkers. SCT: positive BDG result in correlation to the date of stem cell transplantation.

|  |  | Correlation with positive HRCT | | | Correlation with SCT |
| --- | --- | --- | --- | --- | --- |
|  | code | BDG (d) | GM (d) | PCR (d) | BDG (d) |
| PROBABLE IA | K210 | -181, **[17]**, 353 | -3 | (**17**, 32) a | 316, **514**, 850 |
|  | K216 | -35 **to** 45 | **-6 to** 24 | **-6 to -1** | -14 **to** 66 |
|  | K233 | 33 to 47, **66, 79** | neg | (**66, 77**) b | 155 to 169, **188, 201** |
| possible IFD | K235 | [6], 39, 48 | neg | neg | [-12], 21, 30 |
|  | K243 | neg | neg | neg |  |

**K207 – a patient with several suspected infections**

Patient K207 (Fig. 2) was a 12-year-old boy with immunodeficiency in MHC class I complex and a confirmed mutation in the TAP1 gene. His pretransplant medical history was characterized by chronic broncho-pulmonary infections, resulting in a CF-like picture with bronchiectasis and beginning fibrosis. Besides supportive care, regularly immunoglobulins and antibiotics, he was considered eligible for an allogeneic HSCT. After myeloablative conditioning (busulfan/fludarabine/Campath) he received a matched (10/10) unrelated donor transplant with rapid neutrophil engraftment on day 13 and an uneventful course in the early post-transplantation phase. During aplasia standard prophylaxis consisted of voriconazole, later itraconazole. Two months after alloSCT, he developed fever, cough, tachycardia, elevated C-reactive protein (CRP) level and oxygen demand. Thorax radiography revealed progressive infiltrates in the upper lobes compatible with microbial infection. A preexisting *Stenotrophomonas maltophilia* was repeatedly detected in sputum and blood, together with other microorganisms like *Candida* species (in sputum and BAL) and viruses such as CMV and adenovirus in

blood, sputum and BAL. Although the patient partially stabilized under intensified antimicrobial treatment, the further course was characterized by an on-off situation with respiratory deterioration and partial stabilization on an intermediate care unit (IMC). On day 115 he developed an acute septicemia which necessitated mechanical ventilation and circulatory support on an ICU and treatment with voriconazole, meropenem, cefepime and piperacillin/tazobactam (amongst other antibiotics). *Pseudomonas aeruginosa* was cultured from tracheal secretion. Under intensified regimen, the patient recovered partially, but could not be weaned entirely from mechanical ventilation support. On day 219, he died from refractory respiratory distress and multiorgan failure. Interestingly, GM was constantly negative, whereas PCR detected *A. fumigatus* at later stages of the disease between day 114 and 196 post SCT (unfortunately, no samples were available during the phase on the IMC unit). *A. fumigatus* was repeatedly cultured in tracheal secretion from day 194 onwards. HRCT scans were stopped after day 134 as they continued to show severe pulmonary alterations; however, chest X-rays repeatedly documented infectious infiltrates. Due to the lack of HRCT scans in the later phase, the patient was probably wrongly classified as “not classified”, and excluded from overall patient analysis. BDG showed positive results from the first available sample at day 65 post SCT onwards and was intermittently positive until day 114 and positive again at day 196 (next available sample). Multiple infections with *Stenotrophomonas maltophilia* and *Pseudomonas aeruginosa* as pathogens and additionally *A. fumigatus* in the terminal phase are very likely. BDG cross-reactivity with Stenotrophomonas maltophilia and *Pseudomonas aeruginosa* could explain the positivity detected two months after SCT. Also the regular treatment with immunoglobulins is definitely a possible source of elevated BDG levels . For the final and fatal pulmonary infection, *Aspergillus fumigatus* can be regarded as the responsible agent, as this fungus was repeatedly cultured directly from tracheal secretion after day 194 until the death of the patient.

**Figure 2**. Medical history of a patient with several suspected pulmonary infections. Graph displays the course of C-reactive protein (CrP, red) and neutrophils counts (blue) over time, together with the results of the GM, PCR and BDG assays (bars above the graph). For GM, PCR and BDG assays, “red” indicates a positive test result, “green” a negative test result, "light red" an indeterminate result (only for BDG). Antifungal prophylaxis/treatment is outlined in yellow bars below the graph.

**4. Discussion**

The aim of this study was to evaluate a standardized BDG screening (twice weekly) for detection of IA in high-risk pediatric patients. In total, three molecular biomarkers (BDG, GM and *Aspergillus* DNA) were analysed in 404 blood samples of 26 pediatric patients shortly before, during and early after alloSCT. Three cases of probable IA and 2 cases of possible IFD were classified according to EORTC/MSGERC criteria. One not classified patient was excluded and analysed separately. Sensitivity using BDG was high in our cohort (100% including the probable IA patients, 80% including probable IA and possible IFD patients as true IA cases). Our results are in line with previously published results showing a very wide sensitivity range of the Fungitell assay (27% to 100%) . On the other hand the high sensitivity of BDG measurement is hampered by moderate specificity (55%) which again falls into the previously published specificity range of 0% to 100% . Specificity could be improved by requiring a second consecutive positive BDG test or by increasing the cutoff. However, both approaches lead to reduced sensitivity. Adapting the BDG threshold for IFD was discussed for adults , and very recently for pediatric patients . Calitri *et al*. evaluated different cutoffs in children up to 400 pg/ml. Good specificity (>90%) was reached above 200 pg/ml, whereas the best balance between sensitivity and specificity was achieved near 350 pg/ml . This is very similar to the value calculated by our ROC curve analysis (306 pg/ml), but is in clear contrast to BDG cutoffs either reviewed using the EORTC/MSG criteria as a reference standard (60 pg/ml; ) or recommended by the manufacturer (80 pg/ml). A reason for the discrepancy between adult and pediatric cutoff values could be different baseline BDG levels. In fact, Smith *et al*. showed that pediatric patients have a one-third higher background level of BDG in comparison to adults and consequently cutoff values have to be higher in children . Interestingly, for GM a very similar sensitivity and specificity has been reported in adults and children .

The low specificity of BDG is well known and can be caused by several confounding factors including mucositis, bacterial infections or treatment with albumin, immunoglobulins or particular antimicrobial drugs . At least one of these factors was present in each of our patients with false-positive BDG test results but no clear temporal correlation could be established. This proved to be difficult also in other studies . Interestingly, most of the false-positive BDG patients showed clustered positivity around the date of SCT. This is not surprising considering the various treatments the patients receive in this time period. Regular administration of intravenous immunoglobulins that cause significantly elevated BDG levels after a single dose or the development of mucositis with potential translocation of BDG from the gut into the blood stream are just two explanations. As a consequence, careful interpretation of BDG results in this time period is mandatory. Another approach is to exclude BDG completely from screening around the day of SCT. This would increase the specificity of the whole assay tremendously. During this time, clinical signs together with other biomarkers could be used to screen for IFD. According to our data a combination of GM and *Aspergillus*-specific PCR is able to detect probable IA and possible IFD with high specificity (95%) and good PPV (75%). Therefore, GM and *Aspergillus*-specific PCR could be used during the SCT phase. BDG has its place as an additional IFD biomarker after the acute posttransplant phase. Importantly, most of the IFDs occur beyond day 30 after SCT so that the triple combination with BDG, GM and PCR providing the highest sensitivity would probably be optimal in this time.

As BDG is not specific for *Aspergillus*, the test can be used to detect other fungi, but cross-reacts also with some bacteria . Cross-reactivity with microbial antigens could also explain the detection of elevated BDG levels in one possible IFD patient and explain why the more *Aspergillus*–specific assays GM or PCR had not detected this patient. Another case of false-positivity is intensively described above (K207), as this patient suffered from different bacterial infections showing intermittently numerous positive BDG results.

A clear limitation of our study is the limited number of patients in our study cohort resulting in a low number of probable IA cases. As this is a single-center study and only pediatric patients were included, our data represent another brick in the wall of evidences about the applicability of BDG, especially in respect to the pediatric field where data is quite scarce. Our results are in accordance with other published data describing low PPV for BDG screening and therefore limited usefulness as screening tool . On the other hand, BDG can be used as additional diagnostic tool, especially in the post-acute screening phase after alloSCT.

5. Conclusions

BDG can help to diagnose IA in pediatric SCT recipients when used as an adjunctive biomarker especially in the surveillance phase of alloSCT patients after mucositis has resolved and intense antimicrobial treatments are not required any more.

Supplementary Materials: The following are available online at www.mdpi.com/xxx/xy: Figure S1: Matrix of scatterplots indicating the relations between three diagnostic tests. Table S1: Grouping of patients by maxGM index reporting main summary indexes of maxbdg. Table S2. Grouping of patients by maxPCR2019 index reporting main summary indexes of maxBDG. Table S3. Grouping of patients by maxPCR2019 index reporting main summary indexes of maxGM.

**Author Contributions:** Conceptualization, J.S. and J.L.; methodology, J.S., C.M., F.G.; formal analysis, J.S., C.M.; resources, J.H., J.T., O.K., H.E., P.G.S. and M.E..; writing—original draft preparation, J.S, and C.M..; writing—review and editing, J.S., J.H., C.M:, P.G.S., F.G., O.K., H.E., J.L., and M.E.; supervision, J.L.; project administration, J.S., J.L.; funding acquisition, J.H. and J.L.

**Funding**

This study was funded by the German Research Foundation (DFG) through the TRR 124 FungiNet, “Pathogenic fungi and their human host: Networks of Interaction”, DFG project number 210879364, project A2 to HE and JL and project C3 to OK.

**Conflicts of Interest:** J.H. received beta-D-glucan kits free of charge for other studies from Associates of Cape Cod, Inc. and from FUJIFILM Wako Chemicals Europe GmbH. The other authors declared no conflicts of interest.

**References**

1.	**Kedzierska A, Kochan P, Pietrzyk A, Kedzierska J.** 2007. Current status of fungal cell wall components in the immunodiagnostics of invasive fungal infections in humans: galactomannan, mannan and (1-->3)-beta-D-glucan antigens. European journal of clinical microbiology & infectious diseases : official publication of the European Society of Clinical Microbiology **26:**755-766.

2.	**Theel ES, Doern CD.** 2013. beta-D-glucan testing is important for diagnosis of invasive fungal infections. Journal of clinical microbiology **51:**3478-3483.

3.	**Badiee P, Alborzi A, Karimi M, Pourabbas B, Haddadi P, Mardaneh J, Moieni M.** 2012. Diagnostic potential of nested PCR, galactomannan EIA, and beta-D-glucan for invasive aspergillosis in pediatric patients. J Infect Dev Ctries **6:**352-357.

4.	**Liss B, Cornely OA, Hoffmann D, Dimitriou V, Wisplinghoff H.** 2016. 1,3-beta-D-Glucan contamination of common antimicrobials. J Antimicrob Chemother **71:**913-915.

5.	**De Pauw B, Walsh TJ, Donnelly JP, Stevens DA, Edwards JE, Calandra T, Pappas PG, Maertens J, Lortholary O, Kauffman CA, Denning DW, Patterson TF, Maschmeyer G, Bille J, Dismukes WE, Herbrecht R, Hope WW, Kibbler CC, Kullberg BJ, Marr KA, Munoz P, Odds FC, Perfect JR, Restrepo A, Ruhnke M, Segal BH, Sobel JD, Sorrell TC, Viscoli C, Wingard JR, Zaoutis T, Bennett JE.** 2008. Revised definitions of invasive fungal disease from the European Organization for Research and Treatment of Cancer/Invasive Fungal Infections Cooperative Group and the National Institute of Allergy and Infectious Diseases Mycoses Study Group (EORTC/MSG) Consensus Group. Clin Infect Dis **46:**1813-1821.

6.	**Donnelly JP, Chen SC, Kauffman CA, Steinbach WJ, Baddley JW, Verweij PE, Clancy CJ, Wingard JR, Lockhart SR, Groll AH, Sorrell TC, Bassetti M, Akan H, Alexander BD, Andes D, Azoulay E, Bialek R, Bradsher RW, Bretagne S, Calandra T, Caliendo AM, Castagnola E, Cruciani M, Cuenca-Estrella M, Decker CF, Desai SR, Fisher B, Harrison T, Heussel CP, Jensen HE, Kibbler CC, Kontoyiannis DP, Kullberg BJ, Lagrou K, Lamoth F, Lehrnbecher T, Loeffler J, Lortholary O, Maertens J, Marchetti O, Marr KA, Masur H, Meis JF, Morrisey CO, Nucci M, Ostrosky-Zeichner L, Pagano L, Patterson TF, Perfect JR, Racil Z, et al.** 2019. Revision and Update of the Consensus Definitions of Invasive Fungal Disease From the European Organization for Research and Treatment of Cancer and the Mycoses Study Group Education and Research Consortium. Clin Infect Dis.

7.	**Neofytos D, Horn D, Anaissie E, Steinbach W, Olyaei A, Fishman J, Pfaller M, Chang C, Webster K, Marr K.** 2009. Epidemiology and outcome of invasive fungal infection in adult hematopoietic stem cell transplant recipients: analysis of Multicenter Prospective Antifungal Therapy (PATH) Alliance registry. Clin Infect Dis **48:**265-273.

8.	**Hope WW, Denning DW.** 2004. Invasive aspergillosis: current and future challenges in diagnosis and therapy. Clin Microbiol Infect **10:**2-4.

9.	**Barton RC.** 2013. Laboratory diagnosis of invasive aspergillosis: from diagnosis to prediction of outcome. Scientifica (Cairo) **2013:**459405.

10.	**Koltze A, Rath P, Schoning S, Steinmann J, Wichelhaus TA, Bader P, Bochennek K, Lehrnbecher T.** 2015. beta-D-Glucan Screening for Detection of Invasive Fungal Disease in Children Undergoing Allogeneic Hematopoietic Stem Cell Transplantation. Journal of clinical microbiology **53:**2605-2610.

11.	**Bochennek K, Luckowitsch M, Lehrnbecher T.** 2020. Recent advances and future directions in the management of the immunocompromised host. Seminars in oncology **47:**40-47.

12.	**Rogers TR, Morton CO, Springer J, Conneally E, Heinz W, Kenny C, Frost S, Einsele H, Loeffler J.** 2013. Combined real-time PCR and galactomannan surveillance improves diagnosis of invasive aspergillosis in high risk patients with haematological malignancies. Br J Haematol **161:**517-524.

13.	**Aguado JM, Vazquez L, Fernandez-Ruiz M, Villaescusa T, Ruiz-Camps I, Barba P, Silva JT, Batlle M, Solano C, Gallardo D, Heras I, Polo M, Varela R, Vallejo C, Olave T, Lopez-Jimenez J, Rovira M, Parody R, Cuenca-Estrella M, for the Pcraga Study Group the Spanish Stem Cell Transplantation Group tSGoMMotSSoCM, Infectious D, the Spanish Network for Research in Infectious D.** 2014. Serum Galactomannan Versus a Combination of Galactomannan and Polymerase Chain Reaction-Based Aspergillus DNA Detection for Early Therapy of Invasive Aspergillosis in High-Risk Hematological Patients: A Randomized Controlled Trial. Clin Infect Dis.

14.	**Morrissey CO, Chen SCA, Sorrell TC, Milliken S, Bardy PG, Bradstock KF, Szer J, Halliday CL, Gilroy NM, Moore J, Schwarer AP, Guy S, Bajel A, Tramontana AR, Spelman T, Slavin MA, Australasian Leukaemia Lymphoma G, the A, New Zealand Mycology Interest G.** 2013. Galactomannan and PCR versus culture and histology for directing use of antifungal treatment for invasive aspergillosis in high-risk haematology patients: a randomised controlled trial. The Lancet Infectious diseases **13:**519-528.

15.	**Loeffler J, Hafner J, Mengoli C, Wirth C, Heussel CP, Loffler C, White PL, Ullmann AJ, Michel D, Wiegering V, Wolfl M, Schlegel PG, Einsele H, Springer J, Eyrich M.** 2017. Prospective Biomarker Screening for Diagnosis of Invasive Aspergillosis in High-Risk Pediatric Patients. J Clin Microbiol **55:**101-109.

16.	**Springer J, Morton CO, Perry M, Heinz WJ, Paholcsek M, Alzheimer M, Rogers TR, Barnes RA, Einsele H, Loeffler J, White PL.** 2013. Multicenter comparison of serum and whole-blood specimens for detection of Aspergillus DNA in high-risk hematological patients. J Clin Microbiol **51:**1445-1450.

17.	**White PL, Mengoli C, Bretagne S, Cuenca-Estrella M, Finnstrom N, Klingspor L, Melchers WJ, McCulloch E, Barnes RA, Donnelly JP, Loeffler J.** 2011. Evaluation of Aspergillus PCR protocols for testing serum specimens. J Clin Microbiol **49:**3842-3848.

18.	**Springer J, Lackner M, Nachbaur D, Girschikofsky M, Risslegger B, Mutschlechner W, Fritz J, Heinz WJ, Einsele H, Ullmann AJ, Loffler J, Lass-Florl C.** 2016. Prospective multicentre PCR-based Aspergillus DNA screening in high-risk patients with and without primary antifungal mould prophylaxis. Clin Microbiol Infect **22:**80-86.

19.	**Smith PB, Benjamin DK, Jr., Alexander BD, Johnson MD, Finkelman MA, Steinbach WJ.** 2007. Quantification of 1,3-beta-D-glucan levels in children: preliminary data for diagnostic use of the beta-glucan assay in a pediatric setting. Clinical and vaccine immunology : CVI **14:**924-925.

20.	**Goudjil S, Kongolo G, Dusol L, Imestouren F, Cornu M, Leke A, Chouaki T.** 2013. (1-3)-beta-D-glucan levels in candidiasis infections in the critically ill neonate. The journal of maternal-fetal & neonatal medicine : the official journal of the European Association of Perinatal Medicine, the Federation of Asia and Oceania Perinatal Societies, the International Society of Perinatal Obstetricians **26:**44-48.

21.	**De Carolis E, Marchionni F, Torelli R, Angela MG, Pagano L, Murri R, De Pascale G, De Angelis G, Sanguinetti M, Posteraro B.** 2020. Comparative performance evaluation of Wako beta-glucan test and Fungitell assay for the diagnosis of invasive fungal diseases. PloS one **15:**e0236095.

22.	**He S, Hang J-P, Zhang L, Wang F, Zhang D-C, Gong F-H.** 2015. A systematic review and meta-analysis of diagnostic accuracy of serum 1,3-beta-D-glucan for invasive fungal infection: Focus on cutoff levels. Journal of microbiology, immunology, and infection = Wei mian yu gan ran za zhi **48:**351-361.

23.	**Calitri C, Caviglia I, Cangemi G, Furfaro E, Bandettini R, Fioredda F, Amoroso L, Faraci M, Risso FM, Mattioli G, Moscatelli A, Haupt R, Castagnola E.** 2017. Performance of 1,3-beta-D-glucan for diagnosing invasive fungal diseases in children. Mycoses **60:**789-795.

24.	**Metan G, Koc AN, Agkus C, Kaynar LG, Alp E, Eser B.** 2012. Can bacteraemia lead to false positive results in 1,3-beta-D-glucan test? Analysis of 83 bacteraemia episodes in high-risk patients for invasive fungal infections. Rev Iberoam Micol **29:**169-171.

25.	**Buchheidt D, Reinwald M, Spiess B, Boch T, Hofmann WK, Groll AH, Lehrnbecher T, Working Group "Infections in H, Oncology GP-E-S.** 2016. Biomarker-based diagnostic work-up of invasive pulmonary aspergillosis in immunocompromised paediatric patients--is Aspergillus PCR appropriate? Mycoses **59:**67-74.

26.	**Held J, Grawitz AB, Friedrich R, Rappold E, Schulz C.** 2014. Bacteremia by Pseudomonas aeruginosa may cause elevated serum (1 -> 3)-beta-D-glucan levels. Mycoses **57:**33-33.

27.	**Held J, Koch MS, Rappold E, Warnatz K, Schmutz A, Umhau M, Schweizer H.** 2014. Analysis of potential confounding factors causing elevated (1 -> 3)-beta-D-glucan serum levels. Mycoses **57:**32-33.

28.	**White SK, Schmidt RL, Walker BS, Hanson KE.** 2020. (13)-beta-D-glucan testing for the detection of invasive fungal infections in immunocompromised or critically ill people. The Cochrane database of systematic reviews **7:**CD009833.

29.	**Lehrnbecher T, Robinson PD, Fisher BT, Castagnola E, Groll AH, Steinbach WJ, Zaoutis TE, Negeri ZF, Beyene J, Phillips B, Sung L.** 2016. Galactomannan, beta-D-Glucan, and Polymerase Chain Reaction-Based Assays for the Diagnosis of Invasive Fungal Disease in Pediatric Cancer and Hematopoietic Stem Cell Transplantation: A Systematic Review and Meta-Analysis. Clin Infect Dis.

30.	**Held J, Wagner D.** 2011. beta-d-Glucan kinetics for the assessment of treatment response in Pneumocystis jirovecii pneumonia. Clinical microbiology and infection : the official publication of the European Society of Clinical Microbiology and Infectious Diseases **17:**1118-1122.
